# Supplementary material for: Influence of individual models and studies on quantitative mitigation findings in the IPCC Sixth Assessment Report
Source: Nat Commun. 2025 Oct 2;16:8343. doi: 10.1038/s41467-025-64091-w (PMC12491549; doi:10.1038/s41467-025-64091-w)
Supplement: Supplementary file 1 — Supplementary Information [file 41467_2025_64091_MOESM1_ESM.pdf]

Supplementary Information to 'Influence of individual models and studies on quantitative mitigation findings in the IPCC Sixth Assessment Report'

Ida Sognnaes[1]*, Glen P. Peters[1]

[1]CICERO Center for International Climate Research, Oslo, Norway

*Corresponding author: ida.sognnas@cicero.oslo.no

Contents

Supplementary Figures 3
Supplementary Tables 13
Supplementary Notes 18
Supplementary Note 1: ENGAGE study impact on median net-zero GHG year 18
Supplementary Note 2: Differences in variables reporting across models 18
Supplementary Note 3: Challenges in the use of historical data to validate IAMs. 19
Supplementary References 20

Supplementary Figures

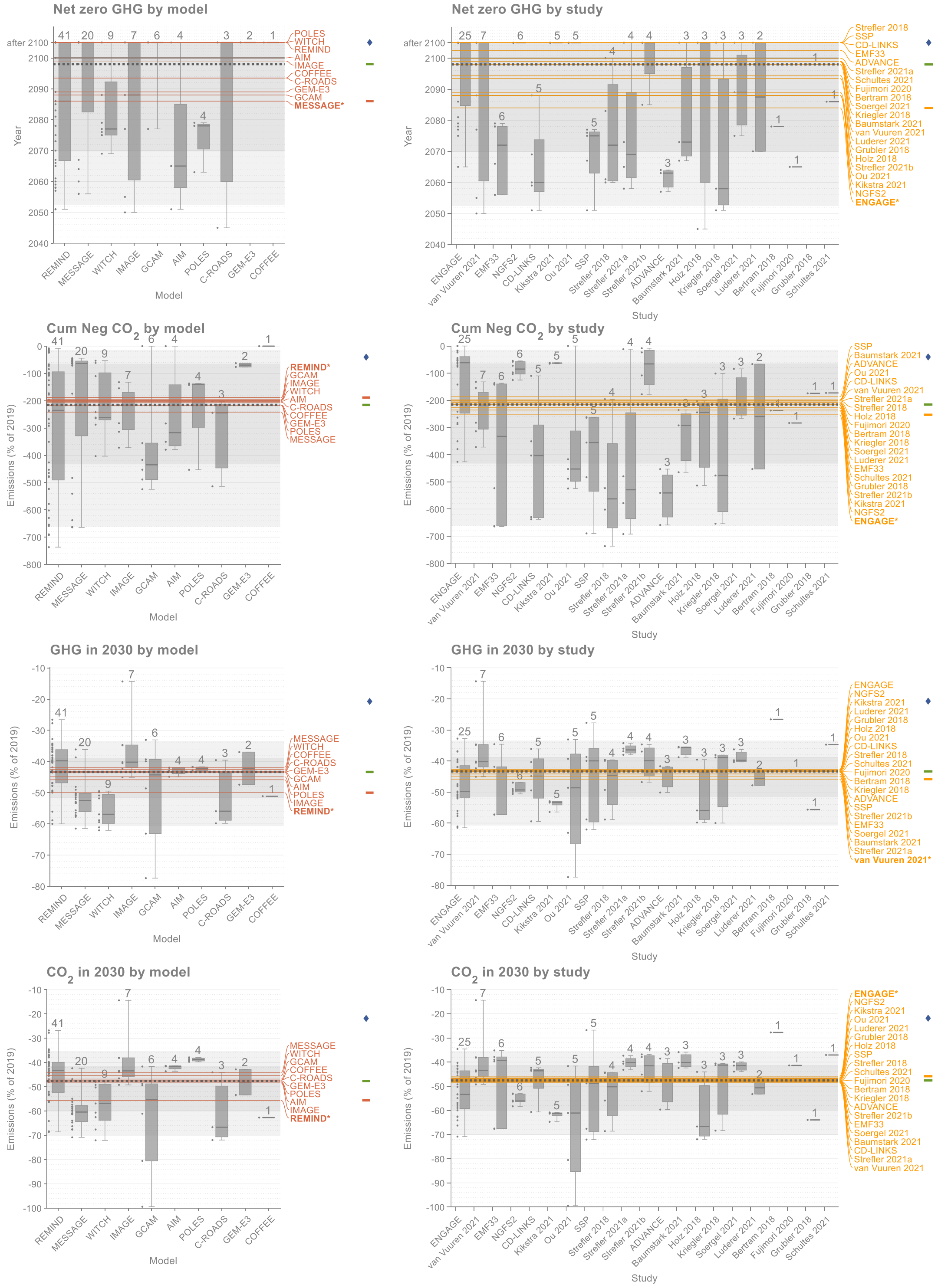

Supplementary Figure 1 Impact of removing individual models and studies on GHG and CO_2 findings. From scenarios that limit global warming to 1.5°C (>50%) (C1 category) reported in AR6 WGIII SPM. Boxes show the minimum, maximum, interquartile range, and median of each model/study. The number of scenarios is shown at the top and data points to the left. Long, horizontal lines show the 1.5°C median (dotted) and the medians when individual models (red) and studies (yellow) are removed. Short, horizontal lines show the 1.5°C median (green), and the median when the model (red) and study (yellow) with the largest impact are removed. The corresponding model/study is bolded and marked with an asterisk. Blue diamonds show the 2°C median. Dark grey patches show interquartile ranges and light grey patches show 5th-95th percentiles. Values on the y-axis above 2100 indicate 'after 2100'. 'Cum Neg CO_2' is cumulative net-negative CO_2 emissions between the year of net zero and 2100. Data: IPCC AR6 Scenarios Database[1].

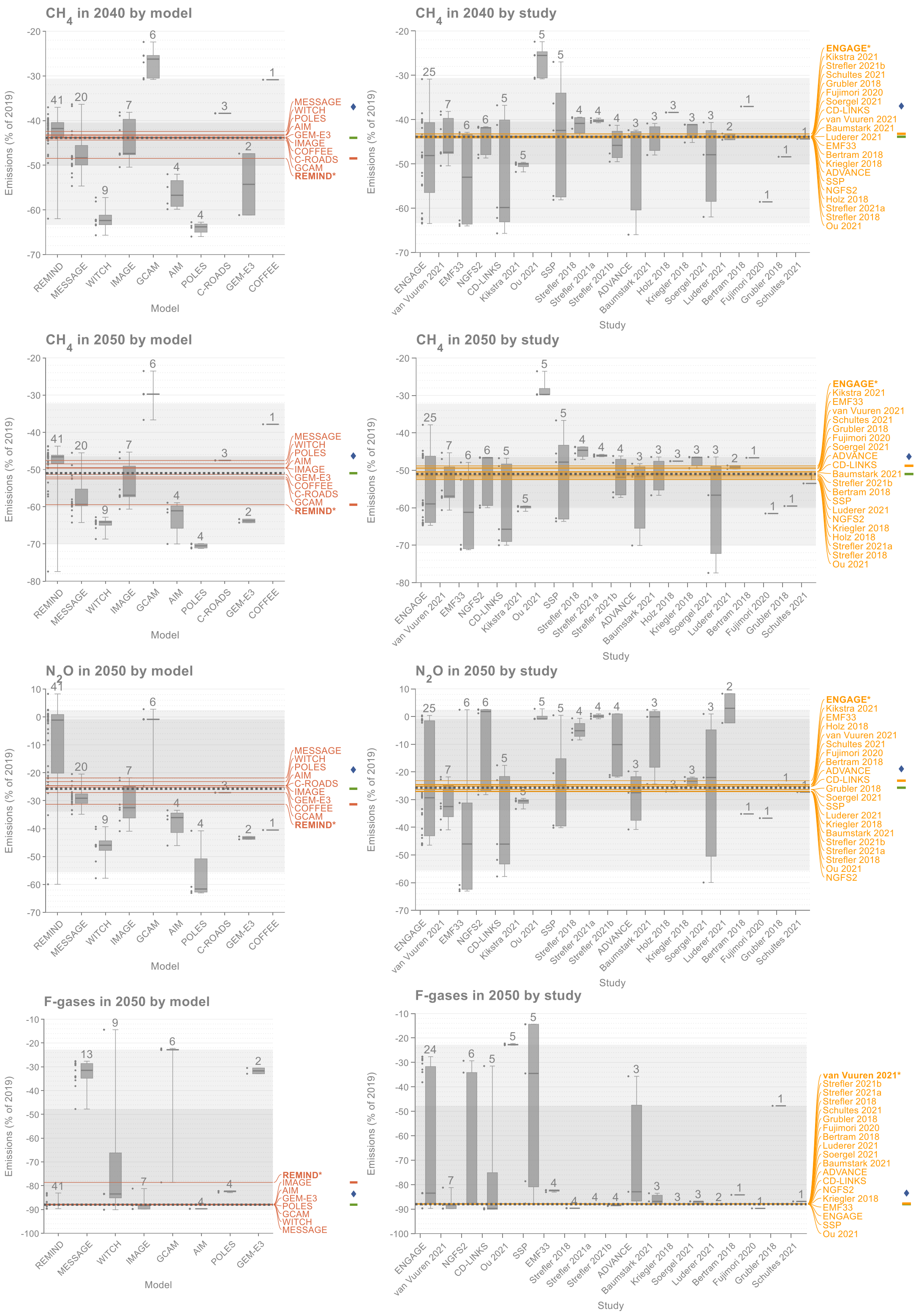

Supplementary Figure 2 Impact of removing individual models and studies on non-CO_2 findings. From scenarios that limit global warming to 1.5°C (>50%) (C1 category) reported in AR6 WGIII SPM. Boxes show the minimum, maximum, interquartile range, and median of each model/study. The number of scenarios is shown at the top and data points to the left. Long, horizontal lines show the 1.5°C median (dotted) and the medians when individual models (red) and studies (yellow) are removed. Short, horizontal lines show the 1.5°C median (green), and the median when the model (red) and study (yellow) with the largest impact are removed. The corresponding model/study is bolded and marked with an asterisk. Blue diamonds show the 2°C median. Dark grey patches show interquartile ranges and light grey patches show 5th-95th percentiles. Data: IPCC AR6 Scenarios Database[1].

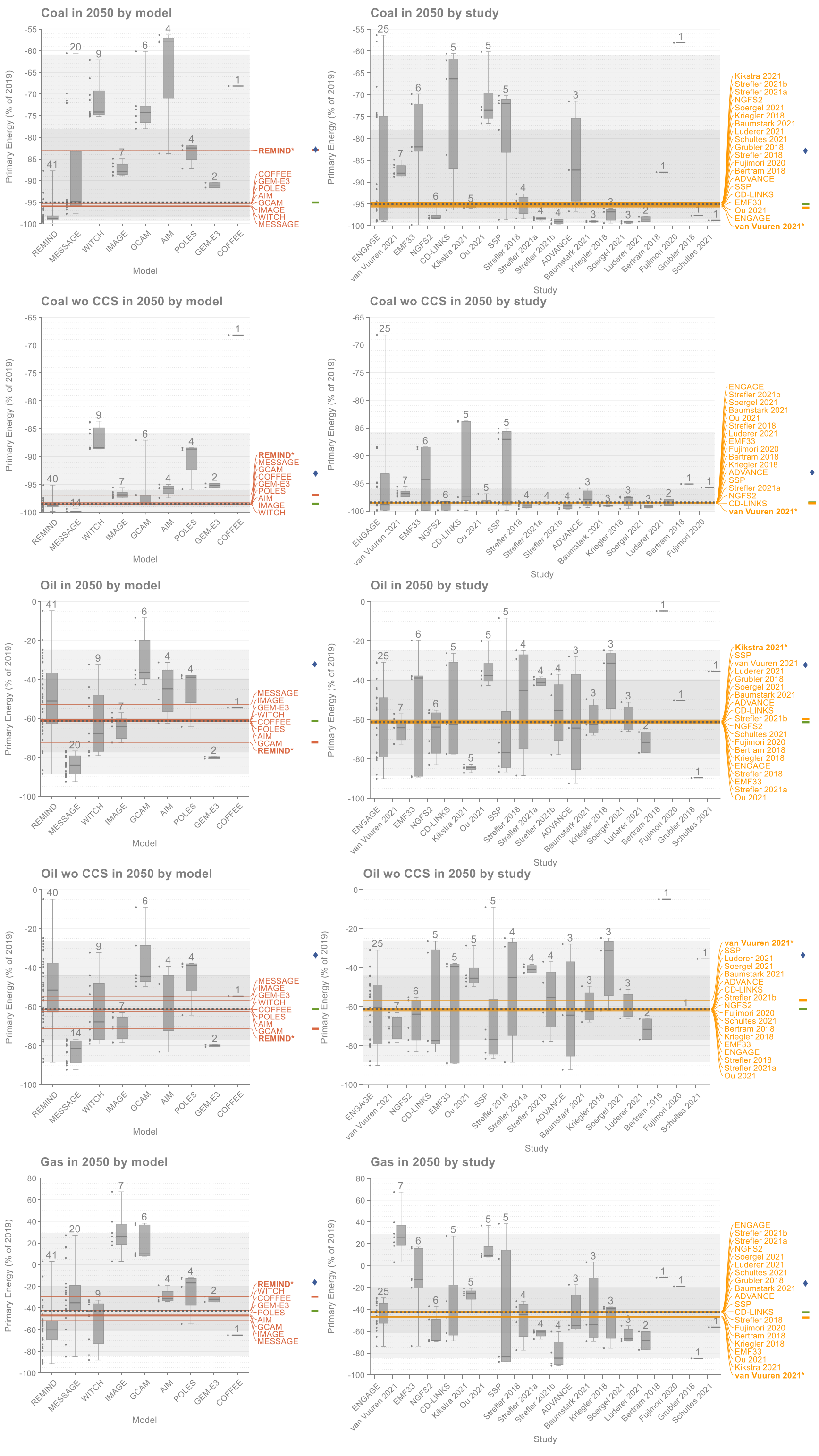

Supplementary Figure 3 Impact of removing individual models and studies on fossil energy findings. From scenarios that limit global warming to 1.5°C (>50%) (C1 category) reported in AR6 WGIII SPM. Boxes show the minimum, maximum, interquartile range, and median of each model/study. The number of scenarios is shown at the top and data points to the left. Long, horizontal lines show the 1.5°C median (dotted) and the medians when individual models (red) and studies (yellow) are removed. Short, horizontal lines show the 1.5°C median (green), and the median when the model (red) and study (yellow) with the largest impact are removed. The corresponding model/study is bolded and marked with an asterisk. Blue diamonds show the 2°C median. Dark grey patches show interquartile ranges and light grey patches show 5th-95th percentiles. 'wo CCS' means 'without Carbon Capture and Storage'. Data: IPCC AR6 Scenarios Database[1].

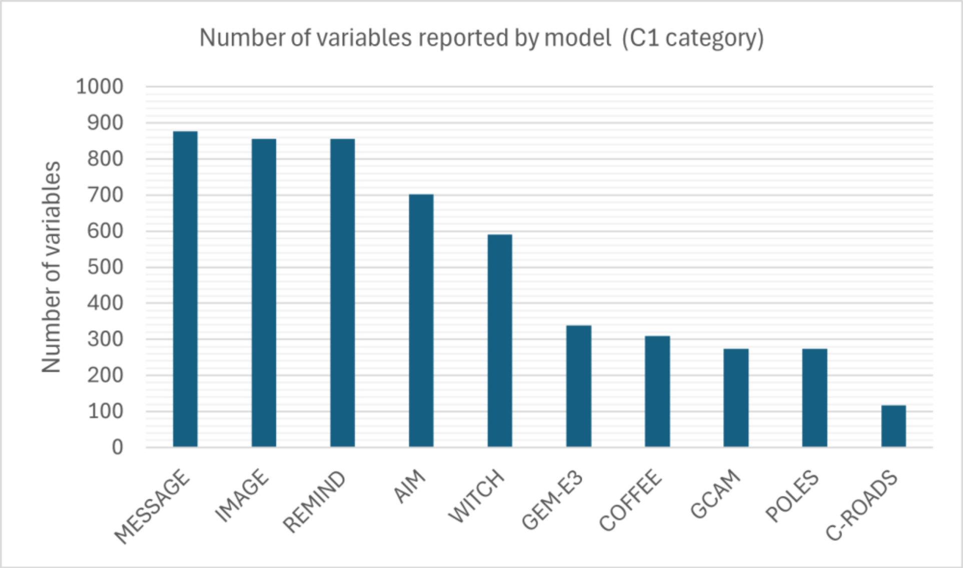

Supplementary Figure 4 Total number of scenario variables reported by each model (across all scenarios) in the C1 climate category (1.5°C scenarios with no or limited overshoot). Only scenarios that passed vetting and received a climate assessment are included. The total number of distinct variables in the database (reported by at least one model in at least one scenarios) is 1442. Data: IPCC AR6 Scenarios Database[1].

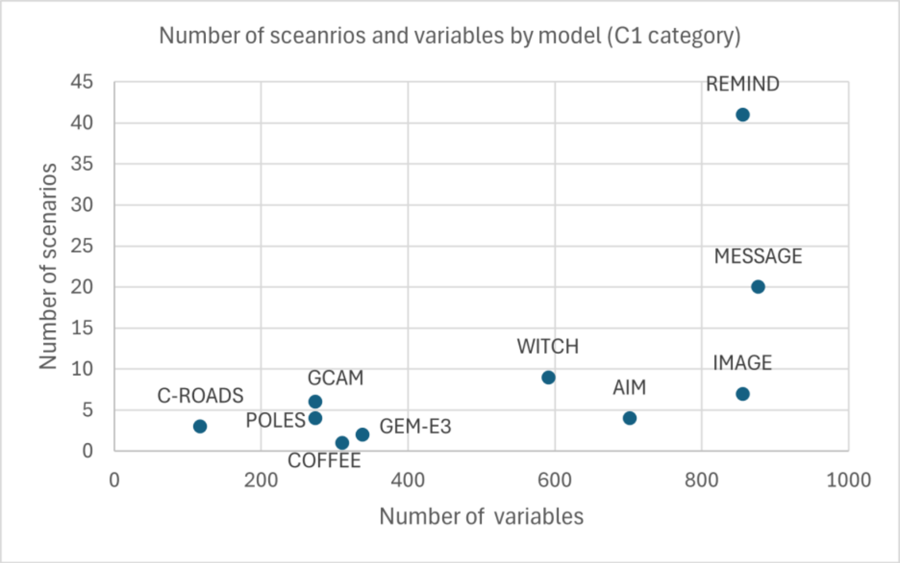

Supplementary Figure 5 Number of scenarios and number of variables by model in the C1 category. Data: IPCC AR6 Scenarios Database[1].

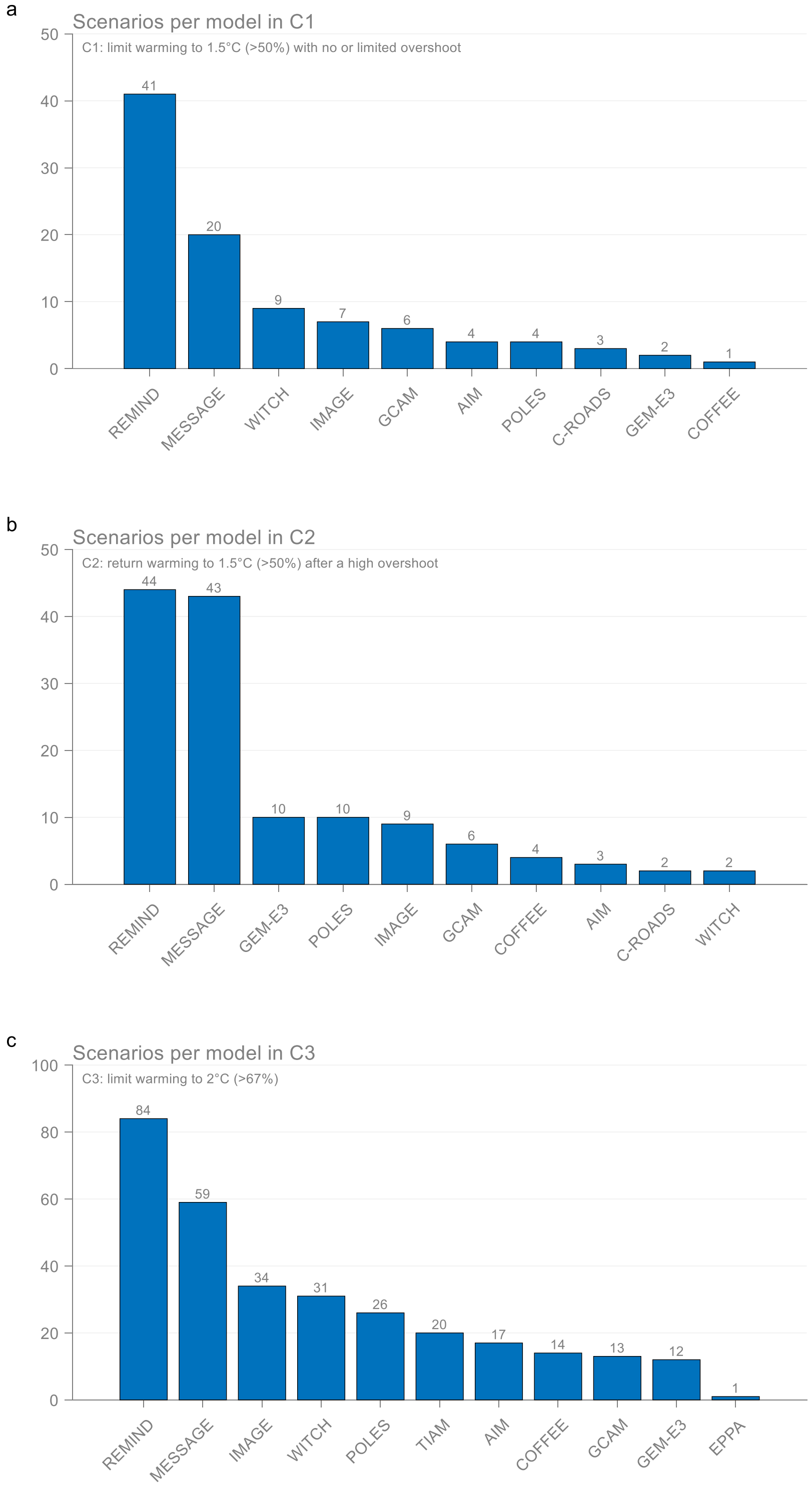

Supplementary Figure 6 Number of scenarios by model in the IPCC AR6 Scenarios Database in the C1 category (**a**), C2 category (**b**), and the C3 category (**c**). Only scenarios that passed vetting and received a climate assessment are shown (97 scenarios in the C1 category, 133 scenarios in the C2 category, and 311 scenarios in the C3 category). The scenario-based mitigation findings in the AR6 WGIII SPM are based on these scenarios. Data: IPCC AR6 Scenarios Database[1].

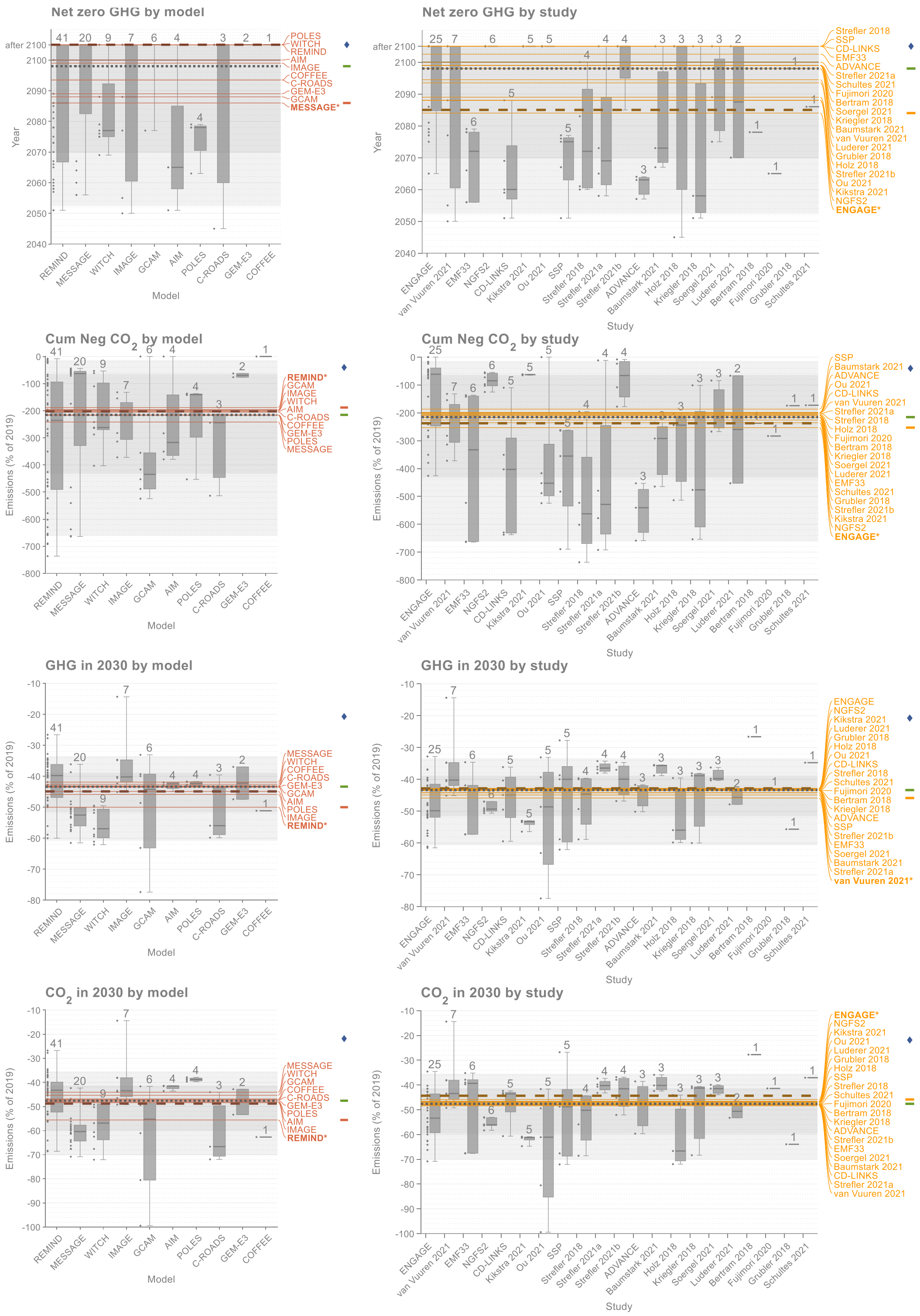

Supplementary Figure 7 Model- and study weighted medians for GHG and CO_2 emissions findings. From scenarios that limit global warming to 1.5°C (>50%) (C1 category) reported in AR6 WGIII SPM. Boxes show the minimum, maximum, interquartile range, and median of each model/study. The number of scenarios is shown at the top and data points to the left. Long, horizontal lines show the 1.5°C median (dotted), the medians when individual models (red) and studies (yellow) are removed, and when weighted by model (red dashed) and by study (yellow dashed). Short, horizontal lines show the 1.5°C median (green), and the median when the model (red) and study (yellow) with the largest impact are removed. The corresponding model/study is bolded and marked with an asterisk. Blue diamonds show the 2°C median. Dark grey patches show interquartile ranges and light grey patches show 5th-95th percentiles. Values on the y-axis above 2100 indicate 'after 2100'. 'Cum Neg CO_2' is cumulative net-negative CO_2 emissions between the year of net zero and 2100. Data: IPCC AR6 Scenarios Database[1].

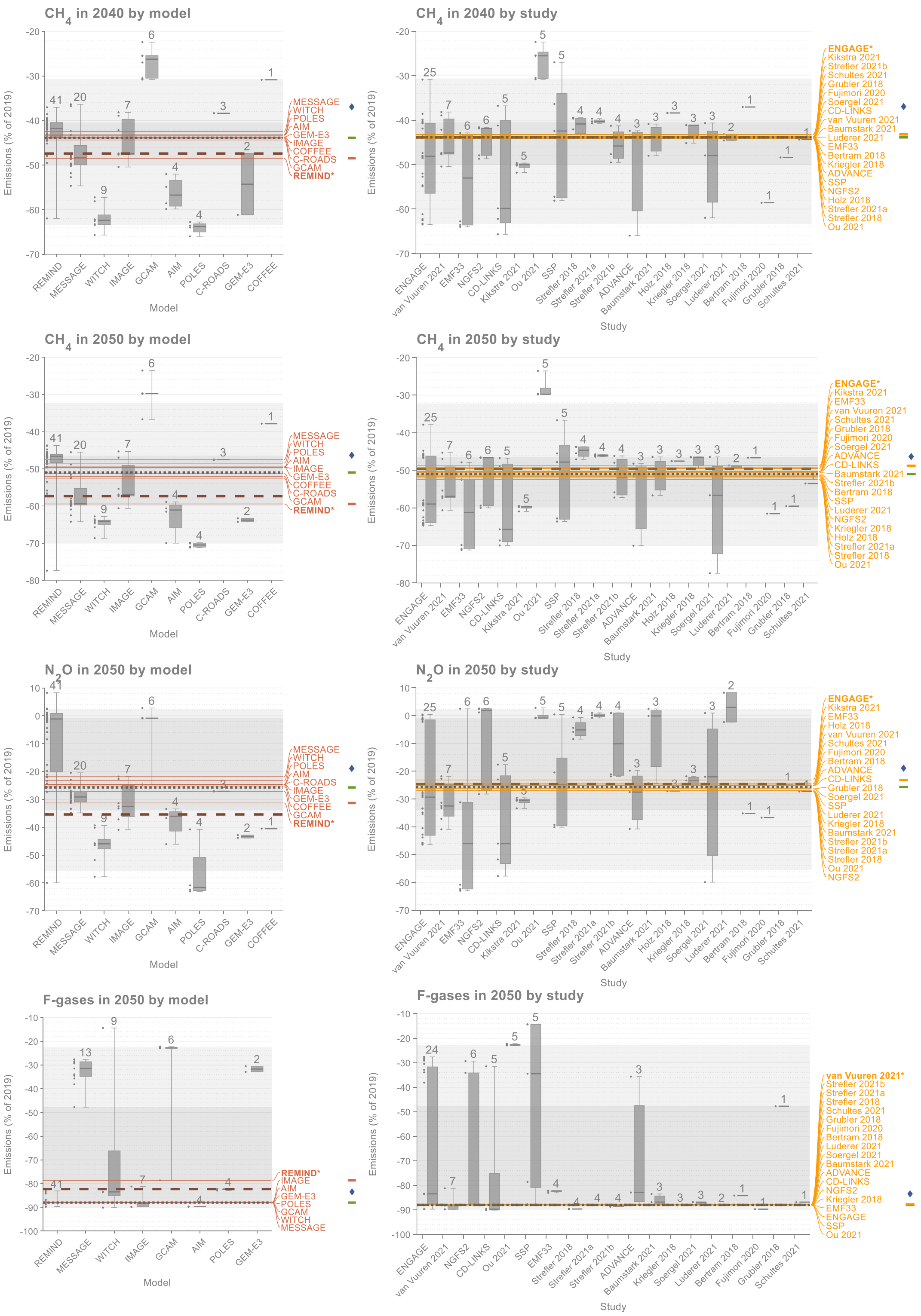

Supplementary Figure 8 Model- and study weighted medians for non-CO_2 emissions findings. From scenarios that limit global warming to 1.5°C (>50%) (C1 category) reported in AR6 WGIII SPM. Boxes show the minimum, maximum, interquartile range, and median of each model/study. The number of scenarios is shown at the top and data points to the left. Long, horizontal lines show the 1.5°C median (dotted), the medians when individual models (red) and studies (yellow) are removed, and when weighted by model (red dashed) and by study (yellow dashed). Short, horizontal lines show the 1.5°C median (green), and the median when the model (red) and study (yellow) with the largest impact are removed. The corresponding model/study is bolded and marked with an asterisk. Blue diamonds show the 2°C median. Dark grey patches show interquartile ranges and light grey patches show 5th-95th percentiles. Data: IPCC AR6 Scenarios Database[1].

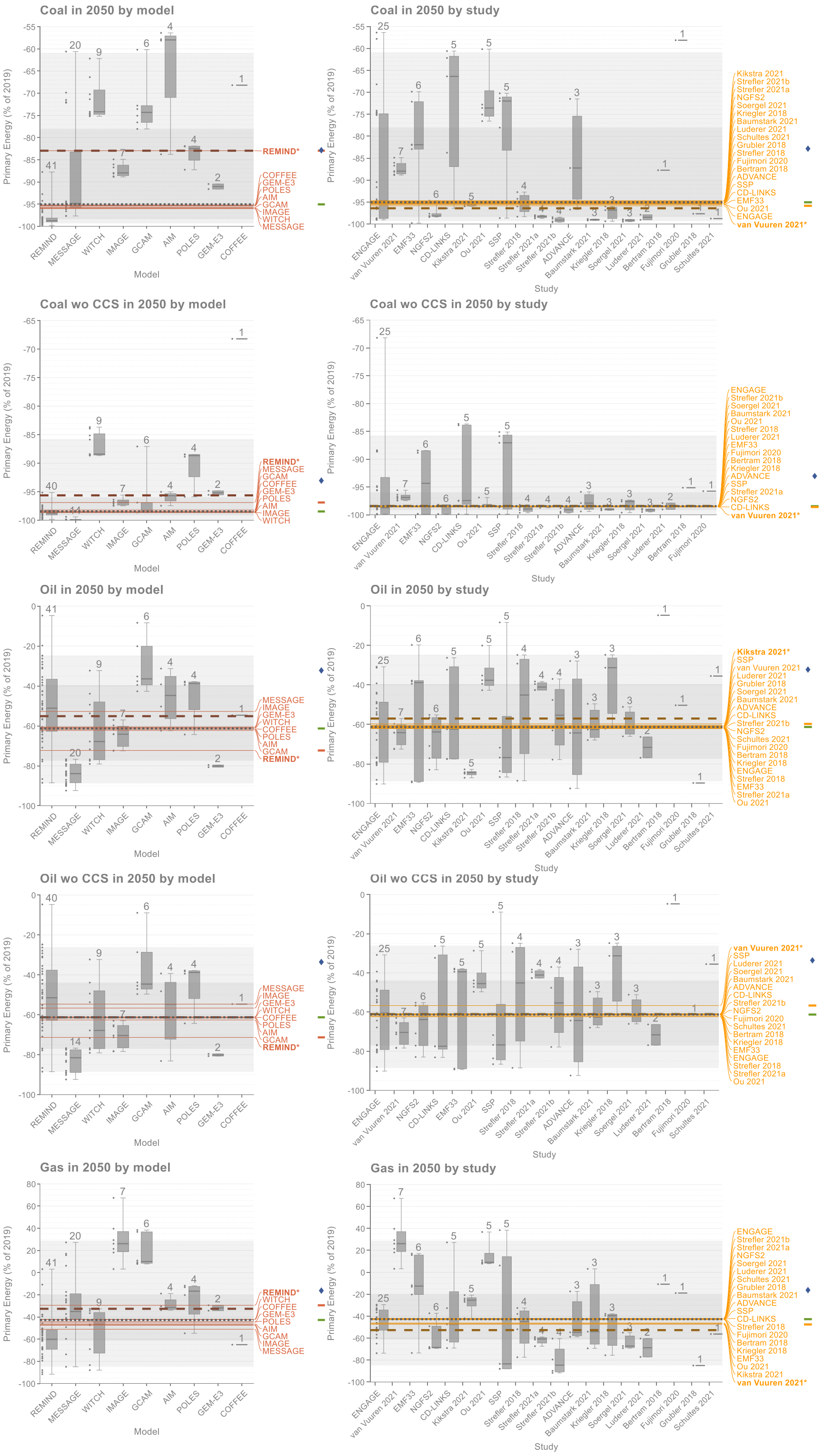

Supplementary Figure 9 Model- and study weighted medians for fossil energy findings. From scenarios that limit global warming to 1.5°C (>50%) (C1 category) reported in AR6 WGIII SPM. Boxes show the minimum, maximum, interquartile range, and median of each model/study. The number of scenarios is shown at the top and data points to the left. Long, horizontal lines show the 1.5°C median (dotted), the medians when individual models (red) and studies (yellow) are removed, and when weighted by model (red dashed) and by study (yellow dashed). Short, horizontal lines show the 1.5°C median (green), and the median when the model (red) and study (yellow) with the largest impact are removed. The corresponding model/study is bolded and marked with an asterisk. Blue diamonds show the 2°C median. Dark grey patches show interquartile ranges and light grey patches show 5th-95th percentiles. 'wo CCS' means 'without Carbon Capture and Storage'. Data: IPCC AR6 Scenarios Database[1]

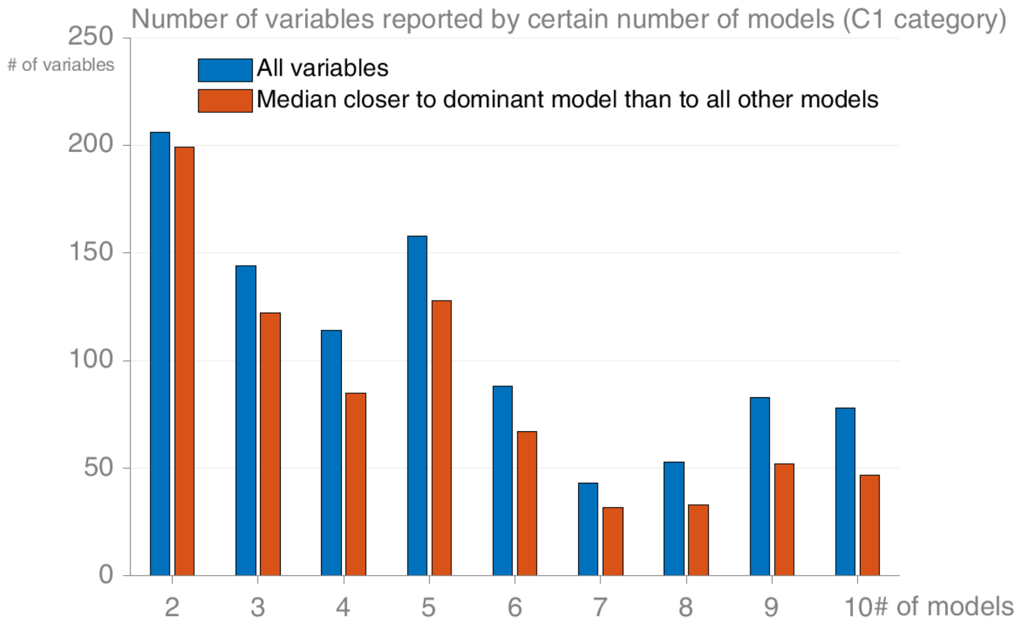

Supplementary Figure 10 Number of variables in the C1 category (1.5°C scenarios with no or limited overshoot) reported by a certain number of models. The total number of variables reported by a certain number of models is shown by the blue bars. The number of variables for which the median is closer to the median of the dominant model than to the median of all the other models is shown by the red bars. Data: IPCC AR6 Scenarios Database[1].

Supplementary Tables

Supplementary Table 1 Impact of removing individual models and studies on median values in the AR6 WGIII SPM, including median values of the dominant model/study and the model/study with the largest impact. The table is sorted according to the 'Between' measure for the models. Numbers are rounded to the nearest digit. Figure 3 shows the top 13 findings that are most impacted according to this measure for either models or studies (values>21%, bolded). Models: R: REMIND, M: MESSAGE. Studies: E: ENGAGE, vV: van Vuuren 2021, K21: Kikstra 2021, S21a: Strefler 2021a, N: NGFS2.

**Scenario findings**[a]		**1.5°C**[b]	**Model remove**[d] **(1.5°C)**					**Study remove**[d] **(1.5°C)**					**2°C**[c]
						Impact measures (%)[e]					Impact measures (%)[e]		
	SPM Source	Median	New Median	Model	Model median	Within	Between	New Median	Study	Study median	Within	Between	Median
F-gases, 2050 (%)	C.1.2	88	79	R	88	99	**207**	88<br>88	vV*<br>E	88<br>83	50<br>1	5<br>1	83
CH_4, 2050 (%)	C.1.2	51	59	R	47	66	**182**	49	E	59	22	**47**	46
Net zero GHG (yr)	Table SPM.2	2098	2086<br>>2100[f]	M*<br>R	>2100[f]<br>2086	63[f]<br>37[f]	**171[f]**<br>**100[f]**	2084	E	>2100[f]	67[f]	**200[f]**	>2100[f]
Coal, 2050 (%)	C.3.2	95	83	R	99	77	**99**	96<br>96	vV*<br>E	88<br>95	10<br>58	7<br>4	83
N_2O, 2050 (%)	C.1.2	26	31	R	1	19	**82**	23	E	29	41	**37**	19
CH_4, 2050 (%)	C.1.2	44	48	R	42	69	**66**	43	E	48	14	10	37
Gas, 2050 (%)	C.3.2	43	29	R	60	43	**50**	48<br>42	vV*<br>E	26<br>43	7<br>69	18<br>3	16
Oil, 2050 (%)	C.3.2	61	72	R	51	52	**38**	60<br>62	K21*<br>E	84<br>61	6<br>25	5<br>1	32
Oil wo. CCS in 2050 (%)	C.3.2	61	71	R	51	51	**36**	57<br>61	vV*<br>E	70<br>61	34<br>17	17<br>1	34
CO_2, 2030 (%)	C.1.2	48	56	R	43	65	**31**	46	E	53	23	7	22
GHG, 2030 ($GtCO_2$-eq/yr)	Table SPM.2	31	28	R	33	69	**30**	30<br>32	vV*<br>E	33<br>28	42<br>13	8<br>4	44
GHG, 2030 (%)	Table SPM.2	43	50	R	40	65	**29**	46<br>43	vV*<br>E	40<br>50	44<br>8	11<br>2	21
Coal wo. CCS in 2050 (%)	C.3.2	98	97	R	99	84	**29**	99<br>98	vV*<br>E	97<br>99	9<br>12	3<br>1	93
Transport-related CO_2, 2050 (%)	C.8.1	59	53<br>62	M*<br>R	62<br>49	64<br>26	19<br>11	57<br>58	vV*<br>E	77<br>60	9<br>8	6<br>0	29

Cumulative net-negative CO_2 ($GtCO_2$)[g]	Table SPM.2	-215	-188	R	236	57	16	-253	E	61	20	**22**	-40
Gas wo. CCS in 2050 (%)	C.3.2	68	64	R	75	41	15	66<br>68	S21a*<br>E	81<br>65	15<br>10	7<br>1	38
Cumulative CO_2 ($GtCO_2$)[h]	Table SPM.2	512	461	R	546	60	14	519<br>515	N*<br>E	449<br>464	10<br>7	2<br>1	888
GHG, 2040 ($GtCO_2$-eq/yr)	Table SPM.2	17	16	R	18	60	13	17<br>17	S21a*<br>E	19<br>17	21<br>69	3<br>3	29
GHG, 2040 (%)	Table SPM.2	69	72	R	66	51	12	70<br>70	S21a*<br>E	66<br>68	22<br>49	4<br>3	46
GHG, 2050 (%)	Table SPM.2	84	86	R	83	57	10	85	E	82	38	6	64
GHG, 2050 ($GtCO_2$-eq/yr)	Table SPM.2	9	8	R	9	54	9	8	E	10	33	5	20
CH_4, 2030 (%)	C.1.2	34	33<br>35	M*<br>R	36<br>33	43<br>46	9<br>8	33	E	36	41	9	19
CO_2, 2040 (%)	C.1.2	80	82	R	76	33	6	79<br>80	N*<br>E	83<br>79	20<br>11	3<br>0	51
Net zero CO_2 (yr)	Table SPM.2	2052	2051<br>2052	M*<br>R	2052<br>2053	100<br>33	5<br>3	2051	E	2055	25	5	2071
Cumulative CO_2 ($GtCO_2$)[i]	Table SPM.2	324	307<br>311	M*<br>R	329<br>339	76<br>47	4<br>3	259	E	384	52	14	797
Peak CO_2 (yr)	Table SPM.2	2020	2020	R	2020	0	0	2020	E	2020	0	0	2020
Peak GHG (yr)	Table SPM.2	2020	2020	R	2020	0	0	2020	E	2020	0	0	2020

[a] Percentages denote reductions from 2019 (negative numbers are increases). Coal, oil, and gas are in primary energy.

[b] 1.5°C (>50%) with no or limited overshoot (C1 category)

[c] 2°C (>67%) (C3 category)

[d] The columns show the median and impact measures when the individual model/study with the largest impact is removed. When the model/study with the largest impact is different from the model/study with the most scenarios, the impact from the latter is shown in the second row. Models and studies that differ from the dominant model and study are marked with an asterisk.

[e] The impacts of removing individual models and studies are measured in two different ways. 'Between' is a unitless measure of the change in median value relative to the difference between the 1.5°C and 2°C medians and 'Within' is a unitless measure of how close the reported median is to the median of the removed model/study versus the median of all the other models/studies. Higher values denote higher impact (see Methods for details).

[f] '>2100' means after 2100. Impact measures may be over- or underestimates because the year can be earlier or much later (the year 2105 is used to calculate the impact).

[g] Between the year of net zero CO_2 and 2100.

[h] Between 2020 and the year of net zero CO_2.

[i] Between 2020 and 2100.

Supplementary Table 2 Models contributing with global scenarios in the AR6 scenarios database and number of scenarios from each model in each scenario category (All, Vetted, Vetted and Climate assessed). The 13 models responsible for the scenarios that passed vetting and received a climate category are written in bold.

**Model Name**	**Number of scenarios in different climate categories**		
	All scenarios	Vetted Scenarios	Vetted with Climate Assessment
**AIM**	**165**	**62**	**55**
BET	16		
C-GEM	32	32	
**C-ROADS**	**6**	**6**	**6**
C3IAM	14	5	
CGE-MOD	32	32	
**COFFEE**	**84**	**65**	**65**
DART	32	17	
DNE21+	49	9	
E3ME	10	10	
EC-MSMR	32	32	
EDF-GEPA	32	32	
EDGE-Buildings	16	8	
ENV-Linkages	15	7	
ENVISAGE	32	32	
**EPPA**	**10**	**7**	**7**
En-ROADS	3		
FARM	13		
GAINS	2	2	
**GCAM**	**157**	**79**	**48**
**GEM-E3**	**52**	**45**	**41**
GEMINI-E3	6	6	
GENeSYS-MOD	1	1	
GMM-17	4	4	
GRAPE	18		
Global TIMES	14		
Global Transportation Roadmap	4	4	
HEB	2		
ICES-EMF	32	32	
ICES-XPS	11		
IEA	1	1	
IIASAPOP	5		
IMACLIM	71	31	
**IMAGE**	**156**	**144**	**142**
LUT-ESTM	1		
MAgPIE	3	3	
**MERGE-ETL**	**3**	**1**	**1**
**MESSAGE**	**297**	**266**	**266**
MIGRATION	10	10	
MUSE	11	5	
McKinsey	3		
**POLES**	**138**	**115**	**114**
PROMETHEUS	7	7	
**REMIND**	**323**	**297**	**297**
REmap	2	2	
SNOW_GL_HH_v1	32	32	
Shell	1		
TEA	32	32	
**TIAM**	**95**	**62**	**45**
WEGDYN	32	32	
WEM	2	2	
**WITCH**	**176**	**115**	**115**
**# Scenarios**	**2297**	**1686**	**1202**
**# Models**	**52**	**41**	**13**

Supplementary Table 3 Model names used in the analysis and corresponding acronyms used in the figures and Table 1. Model names are based on the full model names in the AR6 scenarios database as shown in this table. The unique model names listed here include all models that contributed with at least one scenario that passed vetting and received a climate category. The full model names listed include all the models that contributed with scenarios to the AR6 Scenarios Database, including model versions that contributed with scenarios that did not pass vetting and did not receive a climate assessment.

**Model name (unique)**	**Acronyms used in figures**	**Full model names in AR6 Scenarios Database**
AIM	A	AIM/CGE 2.0, AIM/CGE 2.1, AIM/CGE 2.2, AIM/Hub-Global 2.0
C-ROADS	CR	C-ROADS-5.005
COFFEE	CO	COFFEE 1.1, COPPE-COFFEE 1.0
EPPA		EPPA 6
GCAM	GC	GCAM 4.0, GCAM 4.2, GCAM 5.2, GCAM 5.3, GCAM5.2_NET, GCAM-PR 5.3
GEM-E3	GE	GEM-E3 V1, GEM-E3_V2021,
IMAGE	I	IMAGE 3.0, IMAGE 3.0.1, IMAGE 3.0.2, IMAGE 3.2,
MERGE-ETL		MERGE-ETL 6.0,
MESSAGE	M	MESSAGE V.3, MESSAGE-GLOBIOM 1.0, MESSAGEix-GLOBIOM 1.0, MESSAGEix-GLOBIOM_1.1, MESSAGEix-GLOBIOM_1.2, MESSAGEix-GLOBIOM_GEI 1.0, MESSAGE-Transport V.5
POLES	P	POLES ADVANCE, POLES CD-LINKS, POLES EMF30, POLES EMF33, POLES ENGAGE, POLES GECO2019,
REMIND	R	REMIND 1.6, REMIND 1.7, REMIND 2.1, REMIND_EU 2.0, REMIND-Buildings 2.0, REMIND-H13 2.1, REMIND-MAgPIE 1.5, REMIND-MAgPIE 1.7-3.0, REMIND-MAgPIE 2.0-4.1, REMIND-MAgPIE 2.1-4.2, REMIND-MAgPIE 2.1-4.3, REMIND-Transport 2.1
TIAM		TIAM-ECN 1.1, TIAM-ECN AFR 1.1, TIAM-ECN KEN 1.1, TIAM-ECN ETH 1.1, TIAM-ECN MDG 1.1, TIAM-Grantham 1.0, TIAM-Grantham 3.2, TIAM-UCL 4.1.1, TIAM-WORLD 1.0
WITCH	W	WITCH 4.6, WITCH 5.0, WITCH-GLOBIOM 3.1, WITCH-GLOBIOM 4.2, WITCH-GLOBIOM 4.4

Supplementary Table 4 Study names used in the analysis and corresponding acronyms used in the figures and Table 1. All study names are the same as the 'project_study' given in the AR6 scenarios database metadata.

**Study name (unique) (same as the 'project_study' in the AR6 Scenarios Database)**	**Acronyms used in the figures**
ADVANCE	A
Bauer 2020	
Baumstark 2021	B21
Bertram 2018	B18
CD-LINKS	C
COMMIT	
EMF30	
EMF33	E33
Emmerling 2019	
ENGAGE	E
Fujimori 2020	F20
Giannousakis 2020	
Grubler 2018	G18
Guo 2021	
Holz 2018	H18
Kikstra 2021	K21
Kriegler 2018	K18
Levesque 2021	
Luderer 2021	L21
Marcucci 2017	
NGFS2	N
Ou 2021	O21
PR Policy MIP	
Rottoli 2021	
Schultes 2021	S21
Soergel 2021	So21
SSP	SSP
Strefler 2018	S18
Strefler 2021a	S21a
Strefler 2021b	S21b
Van Vuuren 2021	vV

Supplementary Notes

Supplementary Note 1: ENGAGE study impact on median net-zero GHG year

The ENGAGE study[2] had a goal to explore emission scenarios with less temperature overshoot. To do this the ENGAGE ran two types of mitigation scenarios: 1) a conventional scenario with a 2100 cumulative CO_2 budget that allowed net-negative CO_2 emissions in the second half the century and thereby temperature overshoot, 2) a scenario with the same cumulative CO_2 emissions, but a constraint that required CO_2 emissions to not go below zero, thereby minimising temperature overshoot. The study included nine IAMs and used around 17 cumulative CO_2 budget constraints, and in addition to the two types of mitigation scenarios, included baseline and policy scenarios. Consequently, the study generated hundreds of scenarios, 591 of the 1202 scenarios in the vetted and climate assessed IPCC database.

The ENGAGE scenario design has a direct impact on the year of net zero GHG emissions: Most (not all) of the conventional scenarios reach net zero GHG emissions before 2100, while none of the constrained scenarios achieve net zero GHG emissions. The net zero CO_2 emission year is less impacted, as all scenarios reach net zero CO_2 emissions. They then either continue to net negative CO_2 emissions or maintain net zero CO_2 emissions until 2100. These latter scenarios have positive GHG emissions to 2100 since non-CO_2 emissions are non-zero.

The large number of scenarios from the ENGAGE study, with two very specific scenario designs, in the database explains the large impact on median net-zero GHG year from the ENGAGE study. Hypothetically, if all scenario combinations were run by all models, the distribution of net zero CO_2 or GHG years would be purely bimodal with the median between the two modes. This in itself is a demonstration of the challenge in using statistical distributions of the scenarios database.

Supplementary Note 2: Differences in variables reporting across models

Because different models report different variables (Supplementary Figure 4) and no models report all variables, the model with the most scenarios in the C1 and C3 categories (Supplementary Figure 6), is not the dominant model for every variable (Supplementary Data 1). That different variables are reported by different models has two implications. First, it means that the number of models that report a variable depends on the variable (Supplementary Figure 4). When only a few models report a variable, the impact on the median of the dominant model is generally larger. In the C1 category, many variables are reported by only a few models, and for these variables, the median is almost always closer to the dominant model than to all the other models (Supplementary Figure 10). But even for the variables that are reported by all or most of the models in the C1 category, the median is closer to the median of the dominant model than to the median of all the other models in most cases (Supplementary Figure 10). Second, it means that the number of variables reported by different models plays a role. In general, models that report more variables are likely to be the dominant model for more variables. In the C1 category, for example, IMAGE is the dominant model for more variables than WITCH, even though the two models have a similar number of scenarios (Figure 5, Supplementary Figure 6). This is because IMAGE reports more variables than WITCH and because many of the variables reported by IMAGE are less frequently reported by models with even more scenarios (REMIND, MESSAGE and WITCH), making IMAGE the dominant model for these (Supplementary Data 1). Still, even though MESSAGE and IMAGE both report the same or

more variables than REMIND, the large fraction of scenarios from REMIND in the C1 category (41 out of 97) means that REMIND is the most common dominant model in significantly more cases. In general, models with more scenarios tend to also report more variables, which means that these two effects go in the same direction (Supplementary Figure 5). But overall, the number of scenarios (Supplementary Figure 6) is a better predictor of model dominance (Figure 5) than the number of variables reported (Supplementary Figure 4).

Supplementary Note 3: Challenges in the use of historical data to validate IAMs.
There are multiple limitations associated with the use of historical observations to validate complex models in general[3,4]. Several of these limitations apply to both physical climate models and IAMs[3]: i) a match between historical observations and model simulations might be the result of "forced empirical adequacy", meaning that it is specific to tuned parameters and might not hold in general, ii) because more than one set of parameter values and model structures can yield the same outputs, a match with historical observation is not sufficient to validate the model, and iii) a match with historical observations can be the results of two or several errors that cancel each other out. These limitations imply that a good fit with historical data is not sufficient to guarantee that a complex model accurately represents the modelled system[3].

In addition to the above limitations, IAMs face additional challenges that further limit the ability to validate IAMs using historical observations[3]. First, the modelled system in IAMs may not exhibit structural constancy over time. Socioeconomic and technological processes, which are central to IAMs, are not based on physical principles or laws. Human preferences and political conditions, which determine the relationships between economic variables, technology choice, and emissions in IAMs, are likely to change over time. A good fit with historical data is therefore not a very strong indicator that the model will provide reliable outputs also in the future. Second, most IAMs generate "normative" benchmark scenarios that show how given climate targets can be met in the least-cost manner[5]. Rather than simulating emissions that result from real-world climate policies, most IAMs compute hypothetical least-cost scenarios towards given climate targets. In these scenarios, (cumulative) emissions act as the input (often in the form of a carbon budget), not the output. Even IAM scenarios that explore the impacts of current policies and Nationally Determined Contributions (NDCs) on emissions are hypothetical in the sense that they assume no strengthening of policies beyond current policies and NDC, to act as a point of reference, or benchmark. We do not expect ither of these two scenario types to be realised in the real world. Thus, most IAM scenarios are best understood as hypothetical futures that can be used for 'what-if' analysis. This further limits the potential for historical validation of IAMs.

Despite general limitations ((i)-(iii)), a close fit to observations still builds confidence in a climate models' ability to reliably project climatic responses to future emissions. Historical simulations are therefore key to the validation of climate models[3]. The additional challenges associated with IAMs, however, mean that a close fit to historical observations has only limited relevance for the reliability of IAM outputs under future conditions[3]. In addition to this, there are significant challenges associated with getting historical data for energy and economic systems[3]. For all these reasons, testing of IAMs against historical observations is uncommon. The combination of issues associated with IAMs – a lack of constancy of structure over time, normative scenarios rather than predictions of observed

behaviour, and challenges associated with data collection – together suggest that IAMs cannot be validated using historical data[6].

Supplementary References

1. Byers, E. *et al.* AR6 Scenarios Database. (2022) doi:10.5281/ZENODO.7197970.

2. Riahi, K. *et al.* Cost and attainability of meeting stringent climate targets without overshoot. *Nat Clim Chang* **11**, 1063–1069 (2021).

3. Wilson, C. *et al.* Evaluating process-based integrated assessment models of climate change mitigation. *Clim Change* **166**, (2021).

4. Oreskes, N., Shrader-Frechette, K. & Belitz, K. Verification, Validation, and Confirmation of Numerical Models in the Earth Sciences. *Science (1979)* **263**, 641–646 (1994).

5. IPCC. *Climate Change 2022: Mitigation of Climate Change. Contribution of Working Group III to the Sixth Assessment Report of the Intergovernmental Panel on Climate Change*. (Cambridge University Press, Cambridge, UK and New York, NY, USA, 2022). doi:10.1017/9781009157926.

6. Decarolis, J., Hunter, K. & Sreepathi, S. The case for repeatable analysis with energy economy optimization models. *Energy Econ* (2012) doi:10.1016/j.eneco.2012.07.004.
