# Supplementary material for: Influence of individual models and studies on quantitative mitigation findings in the IPCC Sixth Assessment Report
Source: Nat Commun. 2025 Oct 2;16:8343. doi: 10.1038/s41467-025-64091-w (PMC12491549; doi:10.1038/s41467-025-64091-w)
Supplement: Supplementary file 2 — Description of Addtional Supplementary Files [file 41467_2025_64091_MOESM2_ESM.pdf]

Description of Additional Supplementary Files

Supplementary Data 1 Number of scenarios from each model that report a given variable by climate category (C1-C8) in the AR6 scenario database. 'Total number of scenarios' shows the total number of scenarios (all models) that report a variable in each climate category. 'Number of models that report variable' shows the number of models that report the variable in each climate category. 'Dominant models' shows the dominant model(s) for each variable in each climate category. The dominant model is the model with the most scenarios for a given variable in a given climate category. If more than one model has the most scenarios, all models with the most scenarios are listed. Zero values mean that the variable is not reported by any model. 'Dominant models (>1 model)' shows the dominant model(s) for variables that are reported by a minimum of two models. Zero values mean that the variable is reported by less than 2 models. 'Total variables reported' shows the total number of variables reported by a model (in all scenarios) in each climate category. A value of 0 means the model has no scenarios that passed vetting in this climate category. 'Scenarios per model' shows the number of scenarios from each model in each climate category. 'Dominant model count' shows the number of scenario variables for which the model in question is the dominant model. 'Dominant model count (>1 model)' shows the number of scenario variables, reported by at least two models, for which the model in question is the dominant model. Only scenarios that passed vetting (and received a climate assessment) are included. Data source: Byers, E. et al. AR6 Scenarios Database. (2022) doi:10.5281/ZENODO.7197970.

Supplementary Data 2 Tier 1 and Tier 2 variables in the AR6 scenarios database. Information is based on the AR6 WGIII common scenarios reporting template, available from https://data.ene.iiasa.ac.at/ar6-scenario-submission/#/about.
